# Male allyship in institutional STEMM gender equity initiatives

**DOI:** 10.1371/journal.pone.0248373

**Published:** 2021-03-18

**Authors:** Meredith Nash, Ruby Grant, Robyn Moore, Tania Winzenberg

**Affiliations:** 1 School of Social Sciences, University of Tasmania, Hobart, Australia; 2 School of Social Sciences, University of Tasmania, Launceston, Australia; 3 Menzies Institute for Medical Research, Hobart, Australia; University of Edinburgh, UNITED KINGDOM

## Abstract

This article examines men’s involvement in an institutional gender equity award scheme and how their self-concept as allies develops over time. It draws specifically on a subset of qualitative data from the four men participating in a study involving in-depth interviews with university staff involved in the self-assessment team of one Australian institution’s Science in Australia Gender Equality (SAGE) Athena SWAN pilot. Data related to the men’s experiences is the article’s focus. Key themes from the data include: 1) men’s motivations for engagement; 2) men’s self-understandings as ‘champions for change’ 3) the barriers/risks associated with male championship; and 4) men’s evolving perceptions and critiques of the male champions model. Findings show that men demonstrated personal growth and increased awareness through their participation in the pilot. Yet, their frustration with how equity and diversity was managed in their organisational context highlights pitfalls in the concept of a male ‘champion’. This article provides timely guidance for institutions seeking to engage allies in gender equity initiatives.

## Introduction

Science, technology, engineering, mathematics, and medicine (STEMM) fields are increasingly important contexts for exploring how and why gendered leadership gaps persist [1]. To illustrate, Australian women comprise 50% of science PhD graduates, but only 20% of senior academic leaders [2]. Women comprise 27% of the overall Australian STEMM workforce [3]. Women’s lack of visibility and retention in the Australian STEMM workforce signifies significant gender inequality. To address these trends, in 2016, the Australian Government announced its support for two national institutional gender equity interventions–the Science in Australia Gender Equality (SAGE) program and Male Champions for Change (MCC) STEM. These programs recognise the impact of organisational context on women’s leadership opportunities and pathways. Rather than ‘fixing the women’, these programs aim to ‘fix the system’ [4]. We use the acronym STEMM throughout this article to refer to a broad range of disciplines including Science, Technology, Engineering, Mathematics, and Medicine. In the context of MCC we use STEM (Science, Technology, Engineering, and Mathematics) because this is their acronym of choice. We nevertheless consider that these sections also apply to medicine.

The SAGE program was ‘a response to the Australian Higher Education and Research sector’s need for a coordinated, national approach to improving gender equity in STEMM’ [5]. SAGE’s work is based on the Athena SWAN (AS) Charter for Women in Science–a gender equity award scheme that began in the United Kingdom (UK) in 2005. In 2015, the Athena SWAN Charter was expanded from its original purpose of encouraging structural and cultural change in STEMM organisations and a concomitant increase in the career success of women in STEMM. The Athena SWAN Charter is now focused on gender equity broadly in higher education and research. However, at the time that we conducted this study, SAGE in Australia was focused on gender equity in STEMM, and women specifically. A key component of SAGE’s work is to pilot AS in Australia. Institutions can apply for three AS awards (Bronze, Silver, and Gold) based on progressive levels of institutional and/or departmental performance in recognising and addressing gender inequality [6]. A Bronze award requires an assessment of gender equality, a four-year action plan, and an organisational structure to implement the actions. A Silver award indicates that the institution has successfully implemented the proposed actions and can demonstrate their impact. The Gold award recognises a significant and sustained record of activity and achievement in promoting gender equity within and beyond the institution [7].

At the time of writing, there were 164 AS member organisations in the UK with 962 awards between them [7]. SAGE currently has 42 Australian member institutions with 39 Bronze awards between them [5]. Awards are based on the work of Self-Assessment Teams (SATs)–groups of staff members who collaboratively assess their institution/department against the award criteria and develop the applications. Applications focus on assessing gender equity across the institution, assessing practices and policies in terms of their likelihood of promoting or adversely affecting gender equity, as well as identifying and addressing barriers to gender equality in the organisation. We have previously written about the ways in which gender equity is understood and operationalised by male and female members of one Australian institutional SAT [8]. We identified three key themes related to AS SAT participation and expectations namely: neoliberal understandings of gender equity among SAT members, gender differences in motivations to join the SAT, and corresponding gendered division of SAT labour.

Whereas SAGE is ‘a highly analytical, structured, and long-term programme’, Male Champions of Change (MCC) is ‘disruptive [of the status quo], experimental, and agile’ [4]. Latimer et al. [4] suggest the two initiatives complement each other. MCC was initiated by former Australian Sex Discrimination Commissioner, Elizabeth Broderick, and responds to the challenge of engaging senior men in driving change. In the MCC strategy, ‘decent, powerful men work together to understand gender equality issues and lead action to accelerate progress’ [9]. STEM is now an industry-specific focus area whereby high-profile male leaders ‘influence change and challenge the systems and stereotypes in STEM that hold women back’ [9].

The implementation of MCC STEM demonstrates that allyship is an important component of contemporary efforts to address gendered inequalities in Australian STEMM organisations. Allyship is described in the literature using several terms (e.g. advocate, champion, change agent, sponsor) [10]. Although the definitions vary, broadly, allies align themselves with disadvantaged or oppressed groups and recognise the need for further progress in the journey towards equality [11]. For example, male allies actively confront inequality (e.g. racism, (hetero)sexism) in interpersonal interactions and intervene to address the structural and institutional dimensions of inequality. Crucially, allies recognise their roles in potentially perpetuating the status quo [12]. To be a confident and effective ally, men require a reasonably sophisticated understanding of gendered inequality. Therefore, education about gender equity is integral to the MCC STEM strategy [4,9].

As it relates to the MCC STEM strategy, de Vries [13] notes that the term ‘champion’ emerged to ‘describe a subset of leadership behaviours that focus on the role of executives or senior leaders in relation to change agendas’. This terminology originates in literature supporting the notion that addressing institutional structural inequality is reliant on support from ‘top leaders’ [14]. Interestingly, the term is primarily used in gendered organisational contexts, where men are the main ‘champions’ for gender equality. Thus, an organisational ‘champion for change’ refers to men who have enhanced credibility and positional power to confer approval for a cause, create accountability, and model behaviours and communicate personal diversity experiences that promote that cause over a sustained period [14].

Very seldom is the language of ‘champions’ used for allyship with other equity and diversity causes. For example, white people are not referred to as ‘champions’ for racial equality. Scholars suggest that this is due to the gendered nature of the term ‘champion’ and the way it is deployed in this context. As Kelan and Wratil [14] observe, championing change is entwined with ‘heroic’ masculinity and leadership whereby men ‘take charge’ of inequality. Indeed, the term ‘champion’ has connotations of masculine heroism, Olympic strength and achievement, competitive success, and celebrated goal attainment–all features of a traditional ‘command and control’ style of leadership [15]. ‘Command and control’ leadership is equated with attributes that are socially ascribed to men such as decisiveness, assertiveness, and independence [15]. Given the gendered nature of the term ‘champions’, MCC STEM’s focus on education aims to disrupt routine understandings through listening and learning [4].

There is emerging literature examining the effectiveness of male allyship as an organisational change strategy (e.g. [16]). This is because simply being a ‘good’ man ‘doesn’t make one an ally’ [17]. Rather, a critical element of equity initiatives is that men understand their role as allies. In other words, effective allies understand how to use their powerful positions to support social justice without perpetuating domination [12]. Few studies have examined men’s perceptions of allyship (for an exception, see [10]). Moreover, to our knowledge, none have examined how men learn to become allies over time or how allyship is operationalised in the context of the SAGE program in Australia or elsewhere.

Given their complementary objectives, the SAGE and MCC STEM initiatives provide a valuable context for exploring male allyship in Australian STEMM organisations. This article addresses key knowledge gaps by using qualitative data to examine the extent to which men understand their roles as allies via a qualitative study of a SAGE Athena SWAN pilot program at an Australian university. This pilot did not incorporate MCC STEM’s program. Given our previous findings surrounding the ways in which equity is operationalised and understood in an institutional SAT [8], we became especially interested in men’s perceptions of their personal ally identity development, and their experiences of allyship in the context of the SAGE Athena SWAN gender equity program in the university. First, we review the literature on allyship and its role in addressing organisational change. Next, we describe the methodological framework and findings from the study including a discussion of four key themes including: 1) men’s motivations for engagement; 2) men’s self-understandings as ‘champions for change’ 3) the barriers/risks associated with male championship; and 4) men’s evolving perceptions and critiques of the male champions model. Finally, we discuss the implications of this work.

### Addressing organisational gender inequality through allyship

Several scholars critique the extent to which organisational efforts to address gender inequality take an individualist perspective, rather than acknowledging the structural basis for inequality [18]. For instance, women’s contributions to gendered organisational change have been widely critiqued by feminists (e.g. [19]). This literature emphasises that women and minorities are frequently positioned as the ‘default’ subjects of diversity work in neoliberal organisational contexts. Women of colour especially become ‘ethnographers of universities,’ collecting stories of equity and diversity, inadvertently embodying ‘diversity work,’ by inhabiting bodies assumed to not belong in the white heteropatriarchy of the academy [20]. The unique institutional position of women and minorities often means that undue burden is placed on marginalised people to be ‘diversity poster child[ren]’ who are expected to be committed to equity work within the organisation [20]. This focus on women burdens them with the responsibility for change [21] and obscures the role of formal leadership and power structures. For instance, the default action for many organisations is to encourage women to take leadership development courses to build their capacities, implying that the problem is with the women and not the organisation [1]. In contrast, the feminist organisational change literature emphasises locating gender inequality in the context of collective arrangements and the ‘notable absence’ of men [4].

Given these critiques, greater emphasis is now placed on the need to address men’s roles in gender relations and structures [22]. Because men are senior leaders across all facets of contemporary society, gender equality increasingly requires men’s participation and support [13]. While the role of men in feminist movement and other gender equitable causes is contested, masculinities scholars argue that centring gender inequality on men and male privilege encourages men to take greater responsibility for change [22]. Most of this scholarship originates in anti-violence activism. To illustrate, such approaches can be seen in male bystander programs aiming to combat men’s violence against women [23], men’s pro-feminist and ally groups [24], and formal male ambassador programs and organisations in Australia such as White Ribbon [25]. Anderson [26] contends that several factors affect whether men contribute to equity initiatives. For example, men may be less inclined to participate if they are unaware of their position in a dominant group and the associated gendered privileges [26]. Moreover, men have varying experiences of power–some men do not *feel* powerful despite being associated with patriarchy due to features of identity like social class or race.

Although a large body of scholarship explores a range of social constructions of masculinity [27], one ideal of hegemonic masculinity continues to dominate [28]. In Australia, hegemonic masculinity is traditionally linked with ‘mateship’ in which male identity and relationships are defined by independence, stoicism, and emotional suppression [29]. Thus patriarchy, coupled with the pressure to perform this normative type of masculinity, can affect men’s abilities to become allies. For instance, men can face ‘backlash’ (much like women) if they transgress masculine gender norms and are positioned culturally as ‘weak’ or ‘feminine’ if they publicly support gender equality [30].

Nevertheless, critical analyses of allyship show that male allies can individually benefit from their support of gender equity because they are seen by others as credible and selfless in confronting sexism [12]. This is also an institutional benefit of male allyship because others in an organisation are more inclined to pay closer attention to arguments for gender equality when they are delivered by men. For example, whereas women are often evaluated negatively due to perceptions of self-interest, men are seen as objective in confronting sexism because of the perception that their actions will not benefit them personally [11].

Interest Convergence is a theoretical tool stemming from Critical Race Theory [31]. It describes a situation in which minority rights are only gained when the interests of the marginalised people ‘converge,’ or align, with the mainstream interests of the elite [32]. Interest Convergence is predicated on a sense of ‘my loss-your gain,’ where supporting disadvantaged groups is a ‘loss’ for elites, so equality and diversity measures will only be supported when elites believe there is something to gain [33]. At a structural level, organisational commitment to gender equity and diversity is likely to be embraced when it is seen as being in the interests of the organisation. For example, earning an Athena SWAN award not only enhances a university’s reputation, it is also increasingly linked to research funding. At an individual level, men are more likely to support gender equity initiatives if their interests are met by recognition or reward. Patton and Bondi [12] argue that men often favour activities that provide them with an opportunity to be seen as virtuous, rather than those that may lead to change. However, there are other individual benefits for male allies including bringing other men on board to the cause and modelling a break from traditional masculine gender norms [34]. When men are involved in ally work, men benefit broadly because allyship de-centres idealised masculine gender norms organisationally and makes for more rewarding familial relationships [35].

While their privileged social status enables allies to highlight issues, Patton and Bondi [2] stress that allies ‘should understand how their day-to-day actions, behaviours, and attitudes, resist or perpetuate inequity’. Yet, members of high-status social groups might not recognise discrimination because doing so would undermine the naturalised privileges afforded to their group [36]. To illustrate, men are more likely than women to endorse meritocratic values which position men’s higher social status as rightfully earned [37]. Several studies reveal that men are significantly less likely than women to recognise instances of sexism 11. Similarly, men may have difficulty identifying gendered discrimination, especially when it is subtle or systemic [38]. For example, paternalistic behaviours are often not seen as problematic. Discourses of ‘male championship’ may exacerbate this tendency by suggesting a model of male participation in which men acting on women’s behalf is positive. In contrast, allies are exhorted to collaborate with women, following women’s lead. Given the difficulty men often experience identifying gendered discrimination and understanding their own behaviours, educating male ‘champions’ is a critical component of allyship programs [4].

There is emerging scholarship examining how men perceive their engagement in equity initiatives (e.g. [39]), though this mainly focuses on corporate settings. In her study of professional men involved in gender equality processes in Northern Europe, Kelan [39] identifies three individually focused positions that were aligned with how organisations in the study approached gender equality. The first position is ‘inclusive leader’ where men do personal reflective work to understand their own biases (e.g. implicit bias training) and empathise with others. The second position is ‘smart strategist’ where men focus on the business value of gender equity. The third position is ‘forced altruist’ where legal and/or organisational mandates for social justice force men to sacrifice their own career for the greater good. As Kelan [39] argues, the subject positions problematically construct men as potentially disadvantaged via the organisational focus on gender equality. Furthermore, this model of male allyship does not encourage men to listen and learn from women’s experiences. Rather, these three positions are focused on transforming men into organisationally desired subjects [39]. Only Patton and Bondi’s [12] study specifically centres on men’s experiences of allyship in higher education. This suggests that more information is needed about how organisational context affects men’s allyship activities. For instance, we have limited understanding about what positions men are offered in organisational gender equity efforts and how men take these up. It would also be useful to know if these positions are similar or different depending on organisational context. Similarly, there are few data about how national culture influences the ways that men are engaged in allyship activities (e.g. cross-national comparative studies)–the existing research focuses primarily on allyship in the Anglosphere.

## Materials and methods

### Context

This article draws on a subset of data from a qualitative study of university staff involved in one Australian institution’s SAGE Athena SWAN (AS) pilot between 2017–18. Participants self-selected from the institutional self-assessment team (SAT) composed of 26 male and female academic and professional staff members involved in preparing the University’s Bronze Award application.

### Design

The broader study aimed to explore how AS SAT members’ beliefs and attitudes about gender and gender equity evolved during the AS pilot, their motivations for involvement, and how they experienced SAT membership overall (see Authors 2020). To address the study aims, we conducted interviews with SAT members in years 1 and 2 of the AS pilot. At the time this study was initiated the SAT had 26 members (n = 17 women, n = 9 men) representing 12 [mainly STEMM] disciplines and 5 academic organisational units as well as professional staff members. Twenty-three SAT members were white. SAT members from culturally and linguistically diverse backgrounds were women. The SAT had representation from all university campuses and included members at a range of career points. The leaders of the SAT were STEMM academics. Early in the second half of the first year of the AS pilot all SAT members were invited by email to participate in this study and thirteen (9 women, 4 men) did so.

All SAT members were provided with information about the study and all those who participated provided written informed consent. This article draws specifically on data provided by the four men in the study (see [8] for a summary of women’s experiences). These men were primarily cisgender white senior academic leaders in STEMM fields; three men were Professors and all men held a university, College, or department-level leadership role. Although this is a small sample, it meets the criteria for sample size outlined in Malterud et al. [40] and Morse [41] in relation to aims and scope of the study, study design, analysis strategy and quality of the data. The sample size is also reflective of the context of the specific project focus on one university’s AS SAT.

Two interviews of up to one hour were conducted with each male participant either face-to-face or via phone/Skype. All interviews were conducted using an interview guide. The first interview was conducted between nine and 11 months after participants’ first SAT meeting to assess their experiences of being a SAT member. Participants were asked to describe their professional background, why they applied to be on the SAT, where their ideas about gender equity originate (e.g. What critical episodes/incidents in your life/career have shaped your view of gender equity?), their understanding of the AS pilot process (e.g. Describe what the Athena SWAN pilot means to you? What is the point of the pilot in your view?) and their experiences of the committee over the first year of participation. Participants were also asked to share what they hoped to gain from their participation in the SAT in the second year of the AS pilot.

The second interview, conducted within one year of the first, revisited the discussions from the first interviews, with participants being invited to reflect on the progress of the AS pilot and their involvement in the SAT. For example, participants were asked: How would you describe your working relationship with your committee members? Is there anything about this process that you feel could be improved? What do you feel you have learned about equity and diversity from the data-gathering or self-assessment process? Participants were also asked to share whether any aspects of their knowledge or understanding of gender and gender equality had developed or changed (e.g. How would you explain equity and diversity to a colleague? Has participation in the SAT changed how you feel about equity and diversity (in general)? At the University?). All interviews were audio-recorded and transcribed verbatim with participant consent. This study was approved by the University’s Human Research Ethics Committee. Data in this article have been de-identified and pseudonyms are used.

Researcher identity can shape who participates in social research. In this study, our identities aligned with most of the participants’ as white, cisgender, middle-class professionals. However, we all identify as female and share a feminist commitment to gender equity. As critical researchers, we acknowledge that every aspect of research, from the topics and theoretical paradigms chosen to the interpretations made, are influenced by our standpoints [42]. We are mindful of how the intersecting privileges of whiteness, gender, and class shape the findings of this study. Moreover, Authors 1, 2, and 4 have participated in university SATs and institutional application submissions. This influenced our positioning and access to potential research participants. We acknowledge that while our research questions and methodology, guided by our standpoints, produced one set of findings from many possibilities, the outcomes from this research can inform other gender equity strategies.

### Analysis

To analyse the data from interviews with men we integrated thematic [43] and grounded theory [44] techniques with the aid of QSR NVivo (v.11.2.2 Mac). Thematic analysis is a qualitative technique used to identify meaning within a data set [43]. The thematic analysis was driven by grounded theory, a qualitative methodology that emphasises a systematic inductive approach to data collection and analysis focusing on building theory from data rather than hypotheses [44]. This integrated approach is particularly useful for new areas of study. Grounded theory was chosen because its inductive principles align with the exploratory aims of this research, allowing the team to generate new theories about participation in gender equity award schemes in the Australian context, where little previous research exists. Data were analysed thematically initially by Author 2 first by ‘open coding’, or, surface reading transcripts, taking note of any striking words or phrases arising from the data using NVivo’s annotate function. Once common themes were identified, thematic categories, or ‘nodes,’ were created in NVivo and relevant data was coded to those nodes. To ensure the validity of this thematic analysis and inter-coder reliability of the coding system, Authors 1, 3, and 4 conducted additional analysis and provided critical feedback on the initial interpretation of the data. For this article, data were analysed deductively to specifically explore how male participants discussed their involvement in gender equity and diversity leadership. Any data relating to men’s participation and leadership was initially coded to one deductive parent code and then this data was re-analysed again, allowing inductive themes to emerge. In a final step, using grounded theory, we identified how themes could be correlated and used to develop a conceptual framework for understanding male allyship in the context of the SAGE Athena SWAN pilot.

## Results and discussion

We observed four common themes relating to men’s motivations for involvement in equity and diversity efforts and their understandings of being male champions for change. These include men’s motivations for engagement in gender equity and diversity work; men’s understandings of male championship; the barriers/risks associated with male allyship; and men’s evolving perceptions of allyship.

### Men’s motivations for engagement in gender equity and diversity work

Men became SAT members because they observed gender imbalances in their STEMM disciplines and wanted to contribute by addressing these issues through AS:

There’s nothing that I see as me gaining directly [by participating in the SAT]. I guess indirectly I just see it as the right thing to be doing. Basically because [STEMM field] has a big gender imbalance. So, if you look around my department, men are the largest part of the academic population. I would like to be able to help improve or fix rather than be part of that problem…I was very lucky, I had a supportive family background who let me pursue the sort of things that I was interested in and so I ended up doing my stuff in [STEMM field]. And I think it would be nice if people who had the interest and the aptitude in [STEMM fields] from all sorts of backgrounds were similarly able to have the opportunities that I've had. (Steven, Interview 1)

As a leader in [STEMM department], we have got a challenge in that we’re very male-dominated…We do have some women; not many in the senior levels, and more women are employed on contracts rather than continuing positions. So, there’s a system-based problem with gender equality, which is not good. We’re missing out on that diversity that clearly more gender balance would bring and how that could be much more productive and beneficial…Because the concept of male privilege is that males, including myself, have been privileged by the system that has been in place that has really been a system that has been generated by men. So it’s not surprising that men have been advantaged by this system. (Peter, Interview 1)

These accounts illustrate that men became involved in AS initially because of their leadership roles but draw on personal observations to inform their approaches to SAT membership. In his first interview, Steven regularly referred to the gender imbalance in his field as a primary motivation for wanting to make organisational change. These extracts demonstrate an emerging awareness of gender equity issues as a key driver of participation. These men saw their participation in the SAT as an important opportunity to promote structural change in their institution as well as more broadly in their disciplines. For example, in our study, men identified that gender inequality occurs in STEMM fields and that they (as mostly white men) may be part of the problem. For example, while Steven’s participation in the SAT was based on an ethical stance (‘right thing to do’), he flags his privilege by referring to the ‘luck’ of having many opportunities to build a successful STEMM career. In contrast, Peter is more focused on the benefits that ‘diversity’ brings for the organisation. Peter’s response echoes the broader neoliberal agenda of Athena SWAN programs globally in which women are positioned as an ‘untapped resource in the scientific labour market’ [45]. This pragmatic approach has less in common with the social justice agenda of allies, aligning instead with Kelan’s 39] description of the ‘smart strategist’ position. However, Peter’s reference to male privilege demonstrates his deeper understanding of systemic structural inequality because of participating in the SAT.

### Men’s self-understandings of male championship

In the first year of their engagement with the SAT, the extracts below show that men perceived the label of ‘champion’ as personally empowering and one that encourages men to get involved in social justice work:

One of the things that I took home from the very first session we actually had as a SAT was that [senior leader] told us especially us male participants that we have to be the male champions and I really liked that phrase and that’s what I like to see myself as, as a male champion of this whole process, and wherever I can speak about it I’m quite passionate about it. (Andrew, Interview 1)

I think some of the women [I’ve spoken to] have said it’s great that men are involved in the conversation. My motivation has been that I think men need to be involved in the conversation to be part of the solution, because it’s not a women’s issue, it’s a human issue. (Peter, Interview 1)

I think it's how I can be a champion, supporting women. Not dominating, but how can I walk beside women in the challenges that they face…I think there's a lot of learning. And having to revisit, continually, thoughts on these issues as they come up is important. Some self-reflection, and where I can best support women. I think that internal journey is as important as the external journey. (Rhys, Interview 1)

Rhys articulates an understanding of allyship that is centred on ‘not dominating’ but working alongside women and others in achieving equality. His comments show awareness of the importance of self-reflection and personal growth in allyship. While men in this study appear to be committed to supporting women, they do not discuss specifically how they do this or how they negotiate their own relationships with women in STEMM (see also [12]). For example, Andrew sees himself as a male champion because he identifies the value of passion in disrupting the status quo. Andrew ‘talks the talk’ (e.g. ‘wherever I can speak about it’) but it is not evident how he acts on what he says. Participants are earnest in their desires to make change but largely describe their engagement in change activities in terms of individual effort and not structural change [26].

### Barriers/Risks associated with male allyship

The men in this study received positive feedback from colleagues about their efforts to address institutional gender inequality. For example, in his first interview, Rhys observed:

[I’ve received] just really general support; not too many questions, but [colleagues] acknowledge that it's great that a man is involved in the Athena SWAN, because there's an assumption that…it's only to do with women.

Participants did not appear to have made personal or professional sacrifices at this stage in their path to becoming allies, so this positive feedback is unsurprising. Rather, men are generally praised for doing *something* when most men around them are doing *nothing*. Indeed, the men’s comments are particularly interesting in the context of the gendered labour involved in the SAT itself. As we have discussed elsewhere, women in the broader study referred to the uneven distribution of labour between women and men on the SAT [8]. They also expressed discomfort that men were described as ‘champions’ despite these workload imbalances. However, Peter also observes that a risk of being a male ally is that women may be more suspicious of the process [12]:

I think–it’s interesting, because I think a lot of women, they’re kind of supportive but also suspicious, in whether this is actually going to make a change. Some of the senior females who have been in the system for a long time, they want to see change, but they haven’t seen it in their lifetime. So, I think rightly, so what’s different about this that’s going to make a difference? (Peter, Interview 1)

Peter’s comment indicates that women may be sceptical that a male ally can actually make an impact given historical inactivity in this space, but the comment is also interesting for what Peter does not address–that women and other marginalised groups have much more to lose in advocating for change compared to men like him. Similarly, Peter and other men seem to have less insight into how ‘…their day-to-day actions, behaviours, and attitudes, resist or perpetuate inequity’ (e.g. in the context of the SAT itself) [8,12]. In contrast, Andrew found that some male colleagues were threatened by his involvement in the SAGE pilot, interpreting gender equality efforts as ‘discrimination’ against men:

I don’t want to tread on anybody’s toes and maybe be misinterpreted, but I have a feeling there is still some ice to break and in particular some older male colleagues are probably not really seeing the issue. Some of them are maybe feeling “Is it going too far? By trying to pursue equality is it all of a sudden discriminating against males?” and you do get that little bit of cynical sometimes reluctant feedback which then immediately signals to me “Gee, yes, we do have a huge job here.” (Interview 1)

Andrew identifies a barrier to men’s development of a social justice attitude–recognising privilege [27]. This blind spot profoundly affects the ability of those in privileged groups to understand how oppression works or their own role in perpetuating it. Landreman, Rasmussen, King, and Jiang [46] observe that people in privileged groups have rarely experienced discrimination, so they struggle to recall ‘critical incidents’ that have shaped their awareness of difference. Indeed, men in this study rarely experienced discrimination or had to confront the privilege resulting from multiple aspects of their identities (e.g. race, gender, sexuality) and were unable to identify specific instances of such.

I can’t say that I’m a warrior necessarily for gender equity. I’m a strong believer in equity full-stop. That probably has shaped me from a very early age on. I’ve always been very socially engaged so…for me it’s more about equity be it disability or be it race or Indigenous or religious issues and gender is certainly part of it. (Andrew, Interview 1)

It was brought home to me a lot more what an issue [sexual harassment in STEMM fields] was and how prevalent it was. I guess when it was someone who I did know. So yeah that was pretty upsetting, so I thought it was important to try and ensure that this sort of stuff doesn’t happen and that we address some of these broader issues in academia. (Steven, Interview 1)

By positioning himself as a champion for ‘equity full-stop,’ Andrew emphasises a generic allyship that is not tied to any specific movement politics. Although mentioning gender, disability, race, and religion as intertwined struggles could be interpreted as an intersectional stance, Andrew’s location as a removed supporter for these causes is evident in his statement that he isn’t necessarily a ‘warrior’ for any of them specifically. Moreover, given their limited lived experience of any discrimination, men may be less aware of inequality until it impacts important people in their lives. For example, Steven spoke about how he had abstractly understood gendered discrimination as an issue in STEMM but became deeply aware of women’s experiences when someone he knew was sexually harassed. Here, gender inequality in STEMM became ‘real’ for Steven through listening and learning from women’s lived experiences, motivating him to be an ally.

### Men’s evolving perceptions of allyship

At the beginning of the study, men had less nuanced views about allyship and how they saw themselves as allies. They mainly saw their involvement in the program through their own individual experiences. By the second interview (1 year later), men’s views of their own roles and the wider efficacy of the program had evolved. The extracts below evidence a theme that developed from the second interviews–the notion of ally development as an ongoing process:

So it’s a personal journey I’ve been on for most of my career …And doing Athena Swan, and doing the [data gathering] that I did with [sub-committee], has highlighted, again, the inequities and how far behind universities are, and probably other organisations…there’s this inherent patriarchal society… it’s far more complex than just a single gender equity…And using the terminology that I’ve got to love, you know, intersectionality… and one of my big challenges, was this thing that I call academic privilege, and I call it academic privilege because [laughs]—white privilege is also academic privilege. (Rhys, Interview 2)

So I guess the thing that’s changed for me isn’t so much Athena SWAN…it’s me… And in personal reflections I suppose when I came into this looking at the role of a leader it looked like a job. Coming out the other end I now fully appreciate that leadership is absolutely nothing to do with the job title; it’s a way of being, a way of doing your business. The fact that I lead now in this space has nothing to do with my [leadership] role, and it’s all of our responsibility to lead in this space….I’ve also been realising some of my most irrefutable and ingrained core values are around fairness and equity and social justice. And the things that are most important to me in the way I do business and the way I work with others. So I guess part of that journey of discovery, so it [involvement in the SAGE program] hasn’t changed anything, it’s just made it visible to me about why a lot of the things I do are the way they are because they’re framed in core values of social justice. (Andrew, Interview 2)

The more time has gone on, the more I've realised it is important, as I said, to not just sort of say things, but to actually do things…The Athena SWAN process has made me have to try, and in one sense whenever you think about an issue, you can also come up with a sort of very idealistic “everyone should do this; this is how it should be; this is how we should all change”, but I guess the Athena SWAN process is about actually trying to bring about real change, so that has made me think about the practicalities and also how to do it, how it should be staged and things like that as well. (Steven, Interview 2).

Another important aspect of the second interview was that men described their allyship through action and their accompanying frustration that their efforts were not always successful:

I was asked by my [manager] to help coordinate the implementation of a strategy [for a marginalised group], so…I’ve been very active in promoting that, and nothing has changed… So, I am just embarrassed, because I have tried to push and push this strategy—and it’s on the bottom of the list [for the College]. It’s not even in their consciousness. I could get quite angry there… “Oh, it’s up to the Aboriginal people to do it.” “Oh, it’s up to the gay people to do it.” What about the privileged academics, the privileged whites? We just sit here twiddling our thumbs. (Rhys, Interview 2)

I think the main takeaway that I've had and the main thing that I guess I've learnt probably in the last 12 months is really trying to get a broader engagement in the equity and diversity space. So, last week I went along to a ‘women in science’ event where I was asked to like launch that and, you know, there was like 24, 25 female staff members there and three men. And one of them [men] was me and I was launching it…It should have been about 60, 70 percent men and 30 percent women if it was properly represented. To me, that will be the measure of if we have improved things, we’ll be getting more people to think that “Yes, that hour or so is worth my time”. (Steven, Interview 2)

By the second year of the pilot, men frame their allyship as a continuing practice rather than merely occupying the title of ‘male champions’. In the extracts above, participants situate themselves as part of the solution to organisational inequalities and reflexively acknowledge how they are part of the problem. As Steven says, ‘it’s important…to actually do things’, meaning engage in activities that encourage institutional change. Rhys draws on an intersectional perspective, linking masculinity and white privilege to what he refers to as academic privilege. Rhys consciously implicates himself in the system he is critiquing and the need for meaningful, whole-of organisation change that does not rely solely on the efforts of individuals and/or marginalised groups. Rhys and Steven demonstrate empathy with women and marginalised groups, who they see as only gaining cursory support from colleagues and organisational structures. Importantly, Rhys identifies a flaw in the overarching equity awards process which is that it is mainly focussed on gender equity (see also [45]). As he says, ‘it is far more complex than a single gender equity’.

Rhys and Steven, who had previously been motivated to become allies because of the empowering rhetoric of ‘male championship’, were openly critical of the term and chose to reject it:

I hate the phrase ‘male champions of change’ because it sounds like people putting themselves on a pedestal–‘I'm a champion.’ No, it's actually about being a reasonable person…I think that it's about both being a role model in the sense of just trying to behave the way you would like other people to behave, without being too evangelical about it. (Steven, Interview 2)

So, they wanted men to put up their hands to be male champions, and I get angry about the whole concept. I put my hand up, I even spoke on a panel about my experiences in [my field], and how I felt I could be a male champion, but…we haven’t been supported, there’s been no education, and there were just huge assumptions that we knew what to do to be a male champion. (Rhys, Interview 2)

Given their increased knowledge and self-awareness gained through their AS participation, the participants argue that the gendered positioning of male championship is at odds with gender equity and structural change. Steven does not want to be ‘put on a pedestal’ for being ‘a reasonable person’, while Rhys feels that without formal training the role of a champion is unclear and existing gender norms are reinscribed. As Anderson [26] found in her US study, men’s continuous engagement in equity work is reliant on their satisfaction with their initial experience. In this study, men’s frustration with how equity and diversity was managed in their institution highlights pitfalls in the concept of a male ‘champion’. As these participants’ critiques show, initiatives that empower individual men to speak up about equity and diversity issues take an individualist approach that alone is not enough to address the broader structural issues in organisations. This suggests that when organisations favour more individualist (rather than structural) understandings of leadership, individualist initiatives are prioritised over lasting structural change However, this is problematic when institutions lack clear articulations of diversity and equity because individuals in these settings are then given equally unclear messages about how to enact their roles as allies [12].

## Conclusions

This article addresses critical knowledge gaps in the international literature pertaining to men’s roles in gender equity and diversity efforts in higher education contexts. Building on existing scholarship on allyship and male champions of change, this article explores men’s motivations, experiences, and perceptions of gender equity. This study is especially important because it shifts the dialogue about the labour of equity work from those in underrepresented groups (e.g. women, people of colour, etc.) to men in the most privileged groups. By examining men’s experiences of participating in an AS SAT at an Australian university over the two-year pilot, we identified four key themes including: men’s motivations for engagement in gender equity and diversity work; men’s understandings of male championship; the barriers/risks associated with male allyship; and men’s evolving perceptions of allyship. Findings revealed several ways that participant’s knowledge of gender equity and self-perceptions as allies developed and changed throughout the process. The present study echoed previous research showing that men are more likely to become champions of change if they are aware of gender bias, believe that gender equality is worthwhile, are inclined to defy some masculine gender norms, and are committed to helping others [11].

While participants were initially drawn to the role of male champions, by the second year of the SAGE pilot, most men developed a more critical, intersectional understanding of gender equity and diversity issues, including a sense of uncertainty about the notion of male championship. In this way, the SAGE pilot provided legitimacy for men to embrace their roles as allies and served as an opportunity to be more active in equity initiatives. Although previous research notes that men are more likely to benefit from being seen as allies (e.g. [35]), the men in this study identified that male champions may be seen as having individualistic motivations for their allyship. This demonstrates an unavoidable tension within ally work as allies may be seen as benefiting from their actions. Male allies ‘must continually be reflexive about their positionality’ to avoid reinscribing the gendered status quo while trying to support social justice from a position of power [12]. By their second interview, the men in this study had developed their understanding of their role as allies and in some cases were openly critical of their role. However, little was done at an organisational level to educate and support the male champions in this AS SAT, suggesting that further resources are required to provide meaningful guidance for men in these positions to facilitate substantive organisational change.

A limitation of the self-selected sampling method is that the small sample was biased towards white cisgender men due to the focus on one SAT at one university. As researchers, our own positions as white cisgender women might also have shaped who chose to participate in the research. However, the quality of this study is demonstrated by our in-depth engagement with the topic, sensitivity to context, thorough data collection and methodological transparency, and the in-depth analysis [47]. This study is important because, to our knowledge, there are few studies focusing on male allyship in organisational settings generally and none in relation to the SAGE Athena SWAN program in Australia or elsewhere. The data from this study provides rich evidence of the importance of male allyship and some of the key benefits and challenges associated with taking up this position institutionally. Further research is necessary to examine how men see their role in gender equity work beyond targeted programs like SAGE and how this compares to those working in Athena SWAN programs internationally We also welcome research that examines the experiences of men who opt-out of ally work and the significance of the learning that men in STEMM gain within the context of AS pilots. For instance, the men in our study seemed to have learned a lot in the context of their involvement with the SAGE program which is rarely sold as a ‘benefit’ of engagement. Future research could examine how men’s growth as allies can be optimised within structured equity programs.

### Practical implications

Men’s motivations for becoming allies are diverse and this knowledge should inform strategies to engage men in allyship activities. Institutions should provide diverse opportunities for men to grow as allies through tailored communication that acknowledges that men’s perceptions of allyship and how they might want to engage with that process can and will change over time. Ongoing educational activities are also critical in facilitating male allies’ understanding of core equity issues and the possibilities for allyship in relation to these issues. Finally, regular communication of institutional activities and clear evidence of institutional buy-in is critically important in avoiding a mismatch between individual and institutional efforts. To keep allies engaged, they must believe their efforts matter.
